# Putting Meaning into Meaningful Use: A Roadmap to Successful Integration of Evidence at the Point of Care

**DOI:** 10.2196/medinform.4553

**Published:** 2016-05-19

**Authors:** Thomas McGinn

**Affiliations:** ^1^ Hofstra North Shore LII School of Medicine Manhasset, NY United States

**Keywords:** clinical decision support tools, framework, implementation

## Abstract

Pressures to contain health care costs, personalize patient care, use big data, and to enhance health care quality have highlighted the need for integration of evidence at the point of care. The application of evidence-based medicine (EBM) has great promise in the era of electronic health records (EHRs) and health technology. The most successful integration of evidence into EHRs has been complex decision tools that trigger at a critical point of the clinical visit and include patient specific recommendations. The objective of this viewpoint paper is to investigate why the incorporation of complex CDS tools into the EMR is equally complex and continues to challenge health service researchers and implementation scientists. Poor adoption and sustainability of EBM guidelines and CDS tools at the point of care have persisted and continue to document low rates of usage. The barriers cited by physicians include efficiency, perception of usefulness, information content, user interface, and over-triggering. Building on the traditional EHR implementation frameworks, we review keys strategies for successful CDSs: (1) the quality of the evidence, (2) the potential to reduce unnecessary care, (3) ease of integrating evidence at the point of care, (4) the evidence’s consistency with clinician perceptions and preferences, (5) incorporating bundled sets or automated documentation, and (6) shared decision making tools. As EHRs become commonplace and insurers demand higher quality and evidence-based care, better methods for integrating evidence into everyday care are warranted. We have outlined basic criteria that should be considered before attempting to integrate evidenced-based decision support tools into the EHR.

## Introduction

### Field of Evidence-Based Medicine

**Figure 1:**
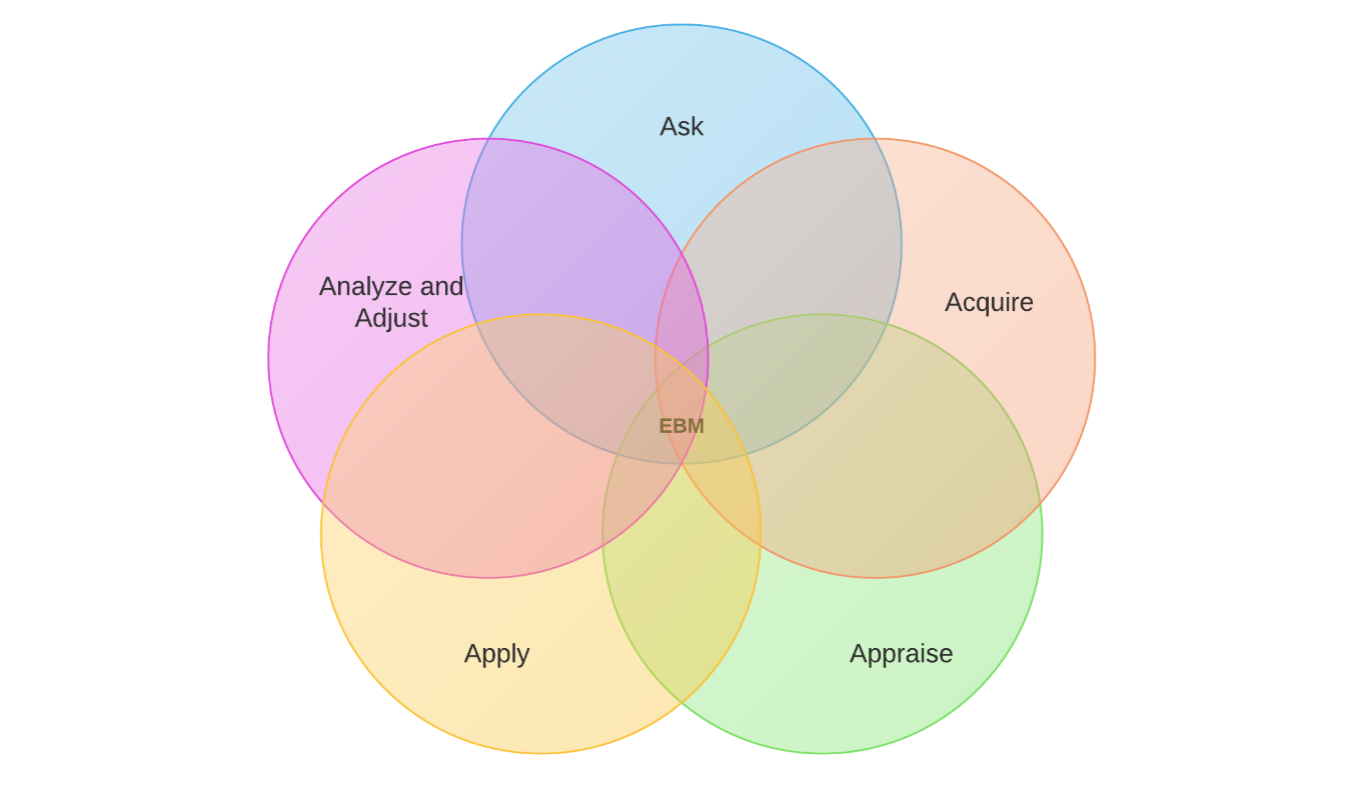
Five steps of evidence-based practice. Evidence-based medicine: EBM.

Pressures to contain health care costs, personalize patient care, use of big data, and enhance health care quality have highlighted the need for integration of evidence at the point of care [1-5]. In the field of evidence-based medicine (EBM), we talk about the evidence cycle (Figure 1 shows this) [6]. The EBM cycle starts with a question (ask), then accessing the evidence (acquire), appraising the evidence, applying the evidence to care for our patients, and analyzing and adjusting [7]. The application step is where researchers and policy makers have struggled with implementation and often failed. Furthermore, the constant evolving evidence-based guidelines, clinical prediction rules (CPRs), and comparative effectiveness results makes it challenging for providers to apply the latest evidence at the point of care. But the direct application of EBM has great promise in the era of electronic health records (EHRs) and health technology.

With the onset of Health Information Technology for Economic and Clinical Health Act and Meaningful Use initiatives in 2009, researchers have been hopeful that health technology will be the solution to bringing EBM to the point of care. Substantial investments of funding, intellect, and energy have yielded an array of EHRs and electronic clinical decision support (CDS) tools to improve patients’ quality of care and reduce inappropriate use of critical resources.

**Figure 2:**
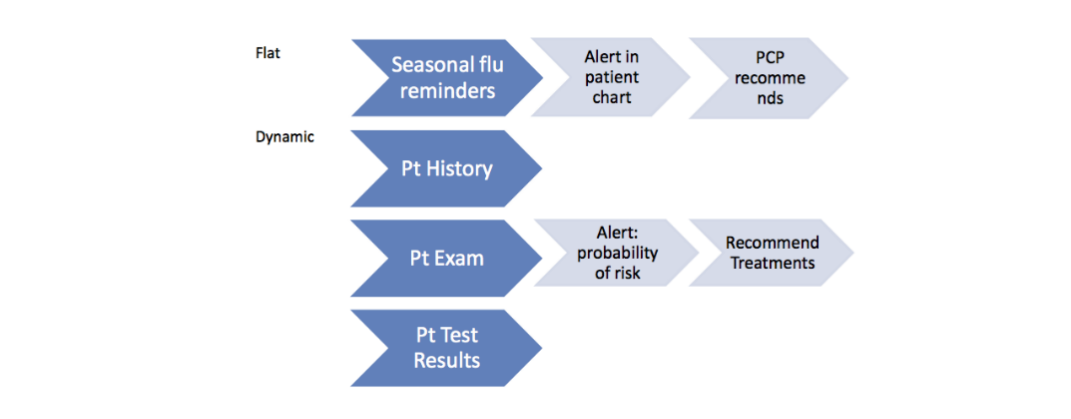
Flat versus dynamic clinical decision support tools. PCP: primary care provider.

The most successful integration of evidence into EHRs has been complex decision tools that trigger at a critical point of the clinical visit and include patient specific recommendations. In contrast, most of the CDS tools being launched are uni-dimensional and not incorporated into the physicians’ workflow. For the purpose of this article, we have designated these forms of evidence integration as “flat reminders”: one-dimensional alerts that are typically triggered by one or two EHR components such as an element of patient history [8-11]. Examples include, flu-shot reminders at annual visits or reminders for colon-cancer screening triggered by patients’ age (Figure 2 shows this). These flat CDS tools unlike complex CDS rarely include patient-specific medical information, are not integrated into the providers’ clinical workflow, do not include tools to support workflow (bundled order sets or documentation corresponding to the tool), or inclusive of patient-centered decision-making tools [12-14].

Complex, multidimensional forms of CDS are patient-specific, provide specific recommendations for rapid frontline decision making, and therefore have had a greater impact on patient outcomes and resource utilization. CPRs are forms of complex CDS. Based on real-time patient data points such as medical history, physical examination, and laboratory data, CPRs are EBM based algorithms that are able to personalize the patient’s diagnosis, prognosis, and likely response to treatment [6]. CPRs weigh patient data and generate a composite score to stratify patients’ risk of disease onset, disease progression, or outcome events. Physicians find these tools more useful, compared to the flat reminders, when decision making is complex, the clinical stakes are high, or cost savings can be achieved without compromising patient care [6]. Adoption of CPRs have been problematic in that applying complex algorithms at the point of care takes additional time, providers’ forget to apply the rule, and they don’t document the usage.

Incorporating complex CDS tools, such as CPRs, into an EHR holds great promise for finally realizing their potential by standardizing their application, reinforcing their application, and documentation. The caveat is that incorporation of complex CDS tools into the EMR is equally complex and continues to challenge health service researchers and implementation scientists. Poor adoption and sustainability of CDS tools at the point of care has persisted and continues to have low rates of usage [15-18]. The barriers cited by physicians include efficiency, perception of usefulness, information content, user interface, and over-triggering [19,20].

**Figure 3:**
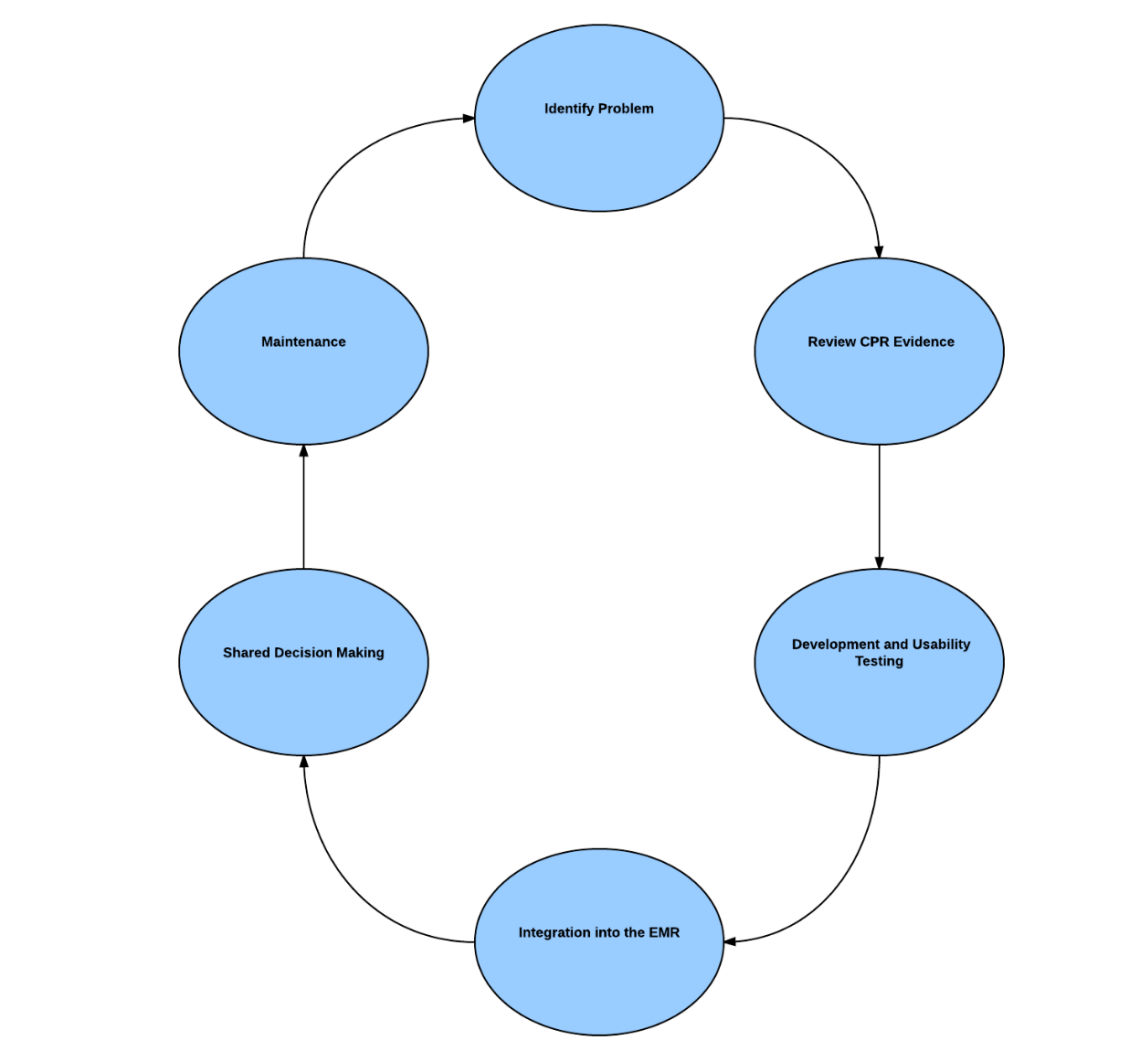
Steps to complex clinical decision support tool integration. CPRs: clinical prediction rules.

Over the past five years, our research team has been working to improve the integration and adoption of complex CDS tools. Similar to the EBM cycle, we see CDS integration as a step wise process of: identifying a clinical problem, reviewing the evidence, usability testing of the tool, integration and deployment of the tool into the EHR, incorporation of shared decision making, and continuous monitoring and maintenance for sustained effectiveness (Figure 3 shows this).

### Aim of the Study

During our research, we have encountered challenges that have repeatedly emerged. In this paper, we propose strategies to overcome those challenges to the integration of complex CDS in order to improve EHR-embedded CDS tools adoption rates, patient outcomes, and resource utilization [21,22].

## Key Considerations for Integrating Evidence-Based Medicine Clinical Decision Support Tools at the Point of Care

### Key Strategies for Successful Clinical Decision Support Tools

Building on the traditional EHR implementation frameworks, we review keys strategies for successful CDSs: (1) the quality of the evidence, (2) the potential to reduce unnecessary care, (3) ease of integrating evidence at the point of care, (4) the evidence’s consistency with clinician perceptions and preferences, (5) incorporating bundled sets or automated documentation, and (6) shared decision-making tools.

### Quality of the Evidence

The first consideration to successful adoption of CDS tools is assessing the quality of the evidence. This may seem an obvious step, but this critical element is often overlooked, with inaccurate assumptions about evidence and impeding the hoped-for results. Therefore, a formal process of evaluating and grading the evidence of the CDS prior to integration is critical. In order for the CDS tool to have a significant impact on health care outcomes, it must be based on high-quality evidence [20].

**Figure 4:**
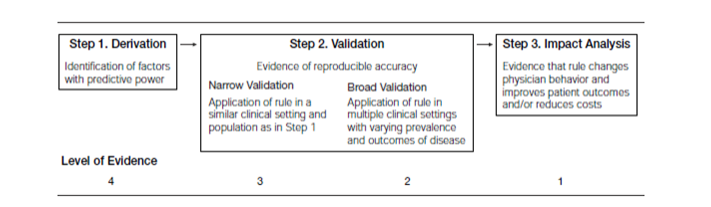
Development and testing of a clinical prediction rule.

The quality of CPRs is determined by how well they have been validated and tested. CPRs are typically developed in a three-step process: (1) derivation of the rule and creation of a model, usually with a retrospective database; (2) validation of the rule, in which the model is tested, preferably in a prospective fashion in several different sites to demonstrate that it is transportable and stable; and (3) impact analysis, when the rule’s impact on clinical behavior is assessed (Figure 4 shows this) [6]. The further along in the development process, the higher the level or quality of evidence and the more ready it is for integration in the EHR. Only CPRs that have reached a level 1 or 2 of evidence and have shown to have a consistent predictive accuracy should be considered for integration.

Several risk-stratification tools with poor and unclear levels of evidence are currently in wide use, for example, the Modified Early Warning Scoring and the pulmonary embolism rule criteria rule for pulmonary embolism. The rules have been derived, but haven’t shown consistency in prospective validations performed in various clinical settings [23-27].

### Potential to Reduce Unnecessary Care

A second consideration we identified as critical is the potential for the evidence to have a significant effect on health care delivery. Historically, CDS and CPR tools have often been introduced in clinical areas plagued by overuse of diagnostic tests or treatments. CPRs aim to accurately identify patients at very low risk who can possibly forgo further testing and those at high risk who can be prioritized for further diagnostic tests or immediate treatment. If the goal of evidence integration is to reduce unnecessary testing or treatment in low risk populations, then estimating how many patients fall into the low risk category will help give an accurate measure of the potential impact of a prediction rule. Our experience has been that estimating this risk will allow you to weigh the potential impact to the work/resources need to build and implement an EHR based CDS tool.

For example, CPRs that guide clinicians through the complex process of risk stratification usually shift the distribution of patients from higher to lower risk. With a CPR such as the strep or pneumonia rule, shifting patients from medium to low risk could reduce orders for antibiotics, which are recommended for patients with medium or high risk; if this constitutes a large proportion of patients, the CPR will have a substantial impact on public health implications (antibiotic resistance) and reduce unnecessary usage of antibiotics.

### Ease of Integrating Evidence at Point of Care

A key to both clinician adoption of CDS tools at the point of care and successful integration is how it easy it is to meld the CPR into workflow and how the patient specific data are entered into the tool. Some CDS tools are extremely complex, requiring multiple data points that may not be automatically integrated into the CDS tool and thus require manual entry. In some practice settings, the EHR may not automatically interface with required data such as x-ray results in the emergency departments or rapid point-of-care test results in primary care clinics. Busy clinicians are unlikely to adopt tools that require them to manually derive, obtain, or enter data. Examples of overly complex tools are the Pneumonia Severity Index (PSI) used in emergency departments to help providers decide to admit patients. The rule has over 20 data elements [28]. Attempts to integrate PSI in emergency room workflow have failed due to poor adoption of the model.

### Automatic, Seamless Triggers

A related issue to ease of integration is “triggering” of the tool. To be truly effective, CDS tools need to be an active (automatic) trigger and seamlessly integrated into the flow of care. Providers should not have to activate the decision support, but rather be automatically offered it in the appropriate setting and related to the appropriate patient. Certain phrases or orders and combinations can act as trigger points in the EMR, such as chief complaint or diagnosis. For a trigger to be successful, it needs to trigger accurately (when truly needed) and not be overly sensitive. In our study on using a CPR for pneumonia, entering cough in the chief complaint section was one method of automatically triggering the complex decision support tool. Ideally, decision support is triggered infrequently and is targeted to the specific condition where it can most assist the provider. If there is no method for accurate triggering, the decision tool may not be effective in changing clinicians’ behavior.

### Consistency of the Evidence With Provider Perceptions and Preferences

Clinicians are most likely to adopt decision support tools or act on evidence guidelines that align with their predispositions about care. Literature suggests providers’ understand the value of CPRs and state they utilize them in decision making, but CPR adoption rates continue to be low and vary across CPRs [15,16,18,29,30]. We have therefore found it helpful to conduct a needs assessment and survey providers on their beliefs and attitudes to better understand their reception and potential for adopting the rules. Furthermore, it allows us to anticipate and approach the cultural barriers to CPR adoption. For example, the success of two accurate CPRs, the Ottawa Ankle Rule (OAR) and the Thrombolysis in Myocardial Infarction (TIMI), varied in clinical impact, not based on the quality of evidence, but upon the attitudes the providers’ had on the utility of the CPR in their practice, which hindered the adoption. Dr Ian Stiell derived and validated the OAR CPR to reduce x-ray ordering in emergency rooms among low risk patients presenting with ankle injuries. Implementation of the rule reduced x-ray ordering by over 30% [31]. In contrast, several prediction rules for chest pain risk stratification in emergency rooms have not been widely adopted despite their demonstrated accuracy [32-34]. Physicians in both examples were presented with accurate CPRs, but behaved differently in each situation. In the case of the OAR, most physicians (89.6%) reported using the rule always or most of the time in appropriate circumstances and 42.2% reported basing their decisions to order radiography primarily on the rule [35]. In contrast, physicians using the TIMI rule reported that they looked at the CPR during the triage in 46% of eligible patients, but only one triage decision (1%) was changed by it [33,36]. The OAR CPR in this situation supports their predisposition to confirm their clinical gestalt and empowers them to follow through. In contrast, patients presenting with chest pain may present physicians with a challenging decision that evidence introduction will not help and therefore evidence alone will not change practice patterns. Performing a needs assessment and survey prior to integrating evidence into workflow will potentially uncover these biases and lead to insight on how to overcome those biases.

### Incorporating Bundled Sets or Automated Documentation

CPRs that can stratify risk and have a corresponding management plan or diagnostic testing, which can be streamlined and bundled into order sets, will likely have more buy in by physicians, leading to higher usage and therefore larger impact on patient outcomes. The largest incentive we have witnessed through our usability testing is how the CPR and CDS tools can streamline clinical practice instead of impeding and slowing it. By incorporating order sets or automated documentation in progress notes of the EMR and automated documentation in progress notes of the EMR, physicians see the CDS as a facilitator rather than a burden. Therefore, we work to develop CDS tools that offer some incentive to using the tool. In our models, we embedded patient education material in both English and Spanish for patients to take home [21,22,37]. We also developed order sets for recommended antibiotics for patients identified as high risk. Both these aspects were popular with providers.

### Shared Decision-Making Tools

The final piece of completing the evidence cycle, which has yet to be sufficiently studied, is the integration of shared decision making when it’s appropriate and as long as it’s based on the best available evidence. Shared decision making (SDM) is becoming an integral part of patient centered care and is seen as a method to improve patient-clinician communication [38,39]. SDM is a process in which the clinician and patient share information about the disease and treatment options and discuss the patient’s preferences to arrive at a decision about a management plan. Decision aids are typically used during the discussions to describe risk of disease and impact of treatment on morbidity and mortality, and have shown to have positive impacts on patient and clinical outcomes [38]. In a systematic review of the literature, it is suggested that patients may benefit from the use of SDM in the emergency department and that SDM is feasible [40]. A randomized controlled trial used SDM tools in patients with chest pain and showed an increase in patients’ knowledge and engagement in decision making and patients decided less frequently to be admitted to the observation unit [41,42]. The combination of CPR with SDM allows for tailored messages around their severity of disease and treatment plans, and through the use of the EMR SDM, reminders, tools, and documentation in clinical visits, CDS is becoming easier.

## Discussion

As EHRs become commonplace and insurers demand higher quality and evidence-based care, better methods for integrating evidence into everyday care are warranted. We have outlined basic criteria that should be considered before attempting to integrate evidenced-based decision support tools into the EHR. First and foremost, this process emphasizes a critical appraisal of the quality of the evidence behind the decision support. Second, CDS tools should be evaluated for their ability to perform and impact clinical care through assessments of providers’ perception of utility. Finally, usability testing and integration into workflow need to be thoroughly evaluated prior to attempts to integrate evidence. Evaluation of the evidence and usability testing, however, are often lacking in research design, implementation methodology, and training of researchers in this area. If the federal government, EHR vendors, or health care institutions do not support research in these areas, the integration of successful CDS tools will continue to lag in creating change in patient outcomes. At this critical juncture of widespread EHRs and pressure to bend the cost curve, incentives to help industry, government, and academic health centers to support these research areas is urgent.

## Abbreviations

CDS: clinical decision support
CPRs: clinical prediction rules
EBM: evidence-based medicine
EHRs: electronic health records
OAR: Ottawa Ankle Rule
PSI: Pneumonia Severity Index
SDM: shared decision making
TIMI: Thrombolysis in Myocardial Infarction
